# VITamin D and OmegA-3 TriaL (VITAL): Effects of Vitamin D Supplements on Risk of Falls in the US Population

**DOI:** 10.1210/clinem/dgaa311

**Published:** 2020-06-03

**Authors:** Meryl S LeBoff, Elle M Murata, Nancy R Cook, Peggy Cawthon, Sharon H Chou, Gregory Kotler, Vadim Bubes, Julie E Buring, JoAnn E Manson

**Affiliations:** 1 Division of Endocrinology, Diabetes and Hypertension, Department of Medicine, Brigham and Women’s Hospital Boston, Massachusetts; 2 Harvard Medical School, Boston, Massachusetts; 3 Division of Preventive Medicine, Department of Medicine, Brigham and Women’s Hospital, Harvard Medical School, Boston, Massachusetts; 4 Department of Epidemiology, Harvard T.H. Chan School of Public Health, Boston, Massachusetts; 5 California Pacific Medical Center, Research Institute, University of California, San Francisco, California

**Keywords:** vitamin D, falls, primary prevention

## Abstract

**Context:**

It is unclear whether vitamin D supplementation reduces risk of falls, and results from randomized controlled trials (RCTs) are conflicting.

**Objective:**

The objective of this work is to determine whether 2000 IU/day of supplemental vitamin D_3_ decreases fall risk.

**Design:**

VITamin D and OmegA-3 TriaL (VITAL) is a double-blind, placebo-controlled RCT including 25 871 adults, randomly assigned November 2011 to March 2014 and treated for 5.3 years (median).

**Setting:**

This is a nationwide study.

**Participants:**

Men 50 years or older and women 55 years or older (mean age, 67.1 years) without cancer or cardiovascular disease at baseline participated in this study.

**Interventions:**

Interventions included vitamin D_3_ (cholecalciferol; 2000 IU/day) and/or omega-3 fatty acids (1 g/day) or respective placebos in a 2 × 2 factorial design.

**Main Outcome Measures:**

Main outcome measures include 2 or more falls and falls resulting in a doctor or hospital visit.

**Results:**

Baseline serum total 25-hydroxyvitamin D (25[OH]D) level was 77 nmol/L; characteristics were well-balanced between groups. Numbers of participants with 2 or more falls were similar between active and placebo groups (9.8% vs 9.4%). Over 5 years, there were no differences in the proportion having 2 or more falls (odds ratio [OR] = 0.97; 95% CI, 0.90-1.05, *P* = .50), falls resulting in a doctor visit (OR = 1.03; 95% CI, 0.94-1.13, *P* = .46), or resulting in a hospital visit (OR = 1.04; 95% CI, 0.90-1.19, *P* = .61) between groups. Results did not differ between those with baseline 25(OH)D less than 50 vs 50 nmol/L or greater or other cut points.

**Conclusion:**

Daily supplemental vitamin D_3_ vs placebo did not decrease fall risk in generally healthy adults not selected for vitamin D insufficiency. This large RCT does not indicate that supplemental vitamin D should be used for primary prevention of falls in the US population.

Falls are a major risk factor for hospitalization and are the leading cause of injury and death from injury in adults age 65 years or older in the United States (1). Each year, an estimated 25% of adults age 65 years or older experience at least one fall (1, 2) and more than 3 million people are treated in emergency departments in the United States for injuries related to falls (3). Recurrent fallers have twice the mortality rate and twice the rate of nursing home admissions compared to one-time fallers (4).

Observational studies suggest an inverse relationship between vitamin D levels and falls. This has been shown in population-based studies among elderly adults (5, 6). In a study of women with hip fractures, those with extremely low vitamin D levels had an increased risk of falls (7). In the Osteoporotic Fractures in Men study in community-dwelling men, low vitamin D levels were associated with frailty at baseline, a risk factor for falls (8). This inverse association of vitamin D and falls in observational studies may be confounded by variables such as nutritional and health status, outdoor physical activity, and other factors.

Systematic reviews and meta-analyses have both supported and recommended against use of vitamin D supplements to prevent falls. In a 2004 meta-analysis of 5 randomized controlled trials (RCTs), Bischoff-Ferrari et al reported that vitamin D supplementation reduced falls among ambulatory and institutionalized older adults by more than 20% (9). In community-dwelling older adults, previous large analyses including those by the US Preventive Services Task Force and Cochrane reported conflicting evidence of effects of vitamin D treatment on reducing fall risk (10-13). A 2014 trial sequential meta-analysis included 20 RCTs of supplemental vitamin D at various doses, from 800 IU/day to 500 000 IU yearly, and concluded that vitamin D supplementation with or without calcium does not reduce falls by 15% or more (14). Other RCTs have found that large boluses of vitamin D (eg, annual bolus of 500 000 IU or monthly bolus of 60 000 IU) resulted in increased falls (15, 16). Studies of effects of supplemental vitamin D on falls are, therefore, conflicting.

Although total 25-hydroxyvitamin D (25[OH]D) is routinely used to assess vitamin D status, it may not be the best biomarker. Vitamin D circulates bound primarily to vitamin D binding protein and, to a lesser extent, albumin. At present, it is unknown whether the free, unbound vitamin D, which represents less than 1% of the circulating vitamin D, may better predict fall risk. We recently found that vitamin D_3_ supplementation compared to placebo did not affect bone density or structure in the VITamin D and OmegA-3 TriaL (VITAL). However, in exploratory analyses, in adults with lower baseline free, but not total, 25(OH)D levels, supplemental vitamin D had a benefit on bone density (17). There are no large RCTs or primary prevention trials of supplemental vitamin D that assess the role of free 25(OH)D levels in predicting the risk of falls.

Given the inconsistencies in previous studies examining effects of vitamin D supplementation on falls, this ancillary VITAL study was designed to determine whether daily, supplemental vitamin D_3_ (2000 IU/d) reduces the risk of falls as assessed by annual questionnaires in the general United States population, and explored whether these effects were modified by baseline levels of total 25(OH)D and/or free 25(OH)D.

## Materials and Methods

### Study design

We investigated effects of daily vitamin D_3_ supplementation (2000 IU/d) on prevention of falls in the VITAL cohort of 25 871 adults (men age ≥50 and women age ≥55 years) recruited by mail, enrolled from 50 US states, and followed for a median of 5.3 years (range, 3.8-6.1 years). VITAL is a recently completed RCT of supplemental cholecalciferol (2000 IU/day) and/or omega-3 fatty acids (1 g/d) vs placebo for primary prevention of cancer and cardiovascular disease (18, 19). There was an oversampling of African Americans (n = 5106). Participants did not have a history of cancer or cardiovascular disease upon trial entry. Safety exclusions included cirrhosis, hypercalcemia, renal failure or dialysis, and other serious conditions (18). Personal use of vitamin D_3_ was limited to 800 IU/day or less and 1200 mg/day or less for calcium (US recommended dietary allowances for older adults) (20). Before random assignment, 39 430 participants entered a 3-month placebo run-in phase, and of these, 13 559 were excluded because of ineligibility, nonadherence, or unwillingness to participate; of these, 25 871 participants were enrolled in VITAL, as summarized in the parent trial paper (18). Random assignment was computer generated within race, sex, and 5-year age groups in blocks of 8. Trial capsules of vitamin D or corresponding placebo (and omega-3 fatty acids or corresponding placebo) were mailed to participants in calendar packs. Additional details on the VITAL protocol have been reported (18, 21, 22). Demographic information and risk factors for falls and comorbidities such as macular degeneration, diabetes, and Parkinson’s disease were assessed through questionnaires at baseline and annually for the study duration. Blood samples were collected in 16 956 at baseline and in 6287 at follow-up among years 1, 2, and 4 (26.8%, 29.5%, and 43.6%, respectively). The trial was approved by Partners Healthcare–Brigham and Women’s Hospital institutional review board and all participants provided written consent.

### Fall measures

Falls were recorded in VITAL questionnaires that were administered at baseline and annually. Falls were defined as “unintentionally coming to rest on the ground, floor, or lower surface.” Participants recorded number of falls (0, 1, 2, or 3+), number of injurious falls defined as falls resulting in limited regular activity for at least one day or in a doctor visit (0, 1, 2, or 3+), and any falls resulting in a hospital visit or being evaluated by a health care provider (yes or no). Two or more falls was selected as the main outcome because it is used to classify those who fall recurrently, which is likely associated with intrinsic rather than extrinsic factors (6). Single falls without injury were not included as the main outcome because a single fall is common among older adults and is most likely coincidental or due to extrinsic factors.

### Physical activity and functional limitation measures

Metabolic equivalent of task (MET) hours were assessed at baseline and 3 years. These tasks included activities such as walking or hiking, jogging, stair climbing, lap swimming, and weight lifting. Participants were asked to fill in the average time per week spent performing such activities, from zero to 7+ hours. Activities of daily living (ADL) and instrumental ADL (IADL) at baseline and years 3, 4, and 5 were assessed by VITAL questionnaires. Analyses included here summarize results from baseline and 5 years.

### Serum measures

Total 25(OH)D levels, including 25(OH)D_2_ and 25(OH)D_3_, were measured by liquid chromatography tandem mass spectrometry (Quest Diagnostics Nicols Institute, San Diego). Free 25(OH)D levels were measured by Future Diagnostics and DIAsource ImmunoAssays S.A. with an enzyme-linked immunosorbent assay 2-step immunoassay, based on patented monoclonal antibody (Wijchen, Netherlands). Precision (% coefficient of variation) for VITAL samples were 4.2% for total 25(OH)D levels and 6.3% for free 25(OH)D levels. Quest Diagnostics performed total 25(OH)D, omega-3 fatty acids, and calcium measurements at no cost to the trial and had no role in study design, analysis, or manuscript writing. VITAL participated in a vitamin D standardization program with the Centers for Disease Control and Prevention (23).

### Statistical analysis

Analyses of effect were based on the intention-to-treat principle. Falls were analyzed as recurrent events over time. We examined the changes in average fall rate from baseline in vitamin D vs placebo groups. Participants without follow-up data were considered missing at random. Fall risk factors were assessed, including body mass index (BMI), history of fracture(s), smoking, alcohol use, medications, baseline calcium and vitamin D supplement use, and physical activity.

Fall rates were determined over time based on the number of people reporting falls in each follow-up year. Fall outcomes were defined as having 2 or more falls the previous year, as injurious falls or those resulting in a doctor visit, and as falls requiring hospitalization. These were modeled with repeated-measures analyses of the proportions over time using generalized linear models with a logit link and robust empirical variance estimates. We used a time by treatment interaction to evaluate the intervention effect, both as the average rates of falling over follow-up as compared to baseline (main intervention effect) and as the trend over time in the intervention effect. We also computed the ratio of odds ratio comparing the average percentage with falls over follow-up compared to baseline in the 2 intervention groups. We analyzed falls during the follow-up in subgroups defined by baseline reports of falls: in groups of no falls, 1 fall, or 2 or more falls in the past year. We further explored interactions between the interventions and we evaluated effect modification by sex, race, age, BMI, and free and total 25(OH)D levels. These vitamin D cutoffs were based on clinical cut points for vitamin D deficiency and insufficiency. The Institute of Medicine recommends 25(OH)D levels 50 nmol/L or greater and suggests less than 30 nmol/L as deficient (24). The Endocrine Society and National Osteoporosis Foundation recommend 25(OH)D levels greater than 75 nmol/L and suggest an estimated 52 to 75 nmol/L as insufficient (25, 26).

Finally, we explored the relationship between treatment assignment and activity (METs), ADL, and IADL outcomes.

Sensitivity analyses for adherence was performed as a secondary analysis by censoring when participants became noncompliant to the use of study agents. All analyses were generated using SAS. Results were considered statistically significant when *P* was less than .05. Because there was no control for multiple hypothesis testing, secondary and subgroup analyses should be interpreted with caution.

## Results

### Study participants

Table 1 shows baseline characteristics of the overall cohort of 25 871 participants. Characteristics of the vitamin D and placebo groups were balanced. Among participants, 51% were women, 20% were black, and 71% were non-Hispanic white. The mean age was 67.1 years. Among participants, 33.3% had at least one fall in the year prior to the trial, 10.0% had a history of fracture, 4.4% had rheumatoid arthritis, 2.7% had a history of macular degeneration, and 24.9% had cataract history. Only 0.3% had Parkinson disease and 0.2% had multiple sclerosis. At baseline, 42.6% were taking vitamin D supplements (≤ 800 IU/d) and 20.0% were taking calcium supplements (≤ 1200 mg/d). Baseline mean serum total 25(OH)D level was 77 nmol/L (n = 16 757) and mean free 25(OH)D level was 15.04 pmol/L (n = 5131).

**Table 1.** Characteristics of participants at baseline, according to random assignment to vitamin D or placebo groups

Characteristic	Total (n = 25 871)	Vitamin D group (n = 12 927)	Placebo group (n = 12 944)	*P*
Female sex, No. (%)	13085 (50.58)	6547 (50.65)	6538 (50.51)	.83
Age, mean (SD), y	67.14 (7.06)	67.13 (7.05)	67.14 (7.08)	.84
Race or ethnic group, No. (%)^*a*^				.96
Non-Hispanic white	18046 (71.32)	9013 (71.27)	9033 (71.37)	
Black	5106 (20.18)	2553 (20.19)	2553 (20.17)	
Nonblack Hispanic	1013 (4.00)	516 (4.08)	497 (3.93)	
Asian	388 (1.53)	188 (1.49)	200 (1.58)	
Native American or Alaskan native	228 (0.90)	118 (0.93)	110 (0.87)	
Other or unknown	523 (2.07)	259 (2.05)	264 (2.09)	
Falls in the past year, No. (%)				0.08
No falls	13256 (66.74)	6561 (66.71)	6695 (67.32)	
One fall	4138 (20.83)	2095 (21.13)	2043 (20.54)	
Two or more falls	2467 (12.42)	1260 (12.71)	1207 (12.14)	
Body mass index, mean (SD), kg/m^2^	28.10 (5.74)	28.12 (5.68)	28.07 (5.79)	.50
Body mass index, No. (%), kg/m^2^				.24
< 18.5	226 (0.89)	113 (0.90)	113 (0.89)	
18.5-24.9	7482 (29.63)	3703 (29.34)	3779 (29.92)	
25-29.9	10097 (39.98)	5047 (39.98)	5050 (39.98)	
30-34.9	4669 (18.49)	2352 (18.63)	2317 (18.34)	
35+	2780 (11.01)	1408 (11.15)	1372 (10.86)	
Parental history of hip fracture, No. (%)	3186 (12.31)	1549 (11.98)	1637 (12.65)	.10
History of fragility fracture, No. (%)	2578 (9.96)	1287 (9.96)	1291 (9.97)	.96
Diabetes history, No. (%)	3549 (13.74)	1812 (14.04)	1737 (13.44)	.16
Use of diabetic medication, No. (%)	2728 (10.55)	1386 (10.73)	1342 (10.37)	.35
Cataract history, No. (%)	6377 (24.87)	3225 (25.17)	3152 (24.57)	.27
Macular degeneration history, No. (%)	686 (2.68)	347 (2.71)	339 (2.65)	.74
Rheumatoid arthritis history, No. (%)	1118 (4.38)	556 (4.36)	562 (4.40)	.87
Multiple sclerosis history, No. (%)	51 (0.20)	24 (0.19)	27 (0.21)	.68
Parkinson disease history, No. (%)	76 (0.30)	45 (0.35)	31 (0.24)	.11
Use of antihypertensive medications, No. (%)	13166 (51.23)	6529 (50.87)	6637 (51.59)	.25
Current use of SSRIs, No. (%)	1621 (6.39)	821 (6.49)	800 (6.30)	.55
Taken an antidepressant or had counseling in past 2 y	2856 (27.37)	1419 (27.51)	1437 (27.24)	.76
Current smoking, No. (%)	1836 (7.20)	921 (7.24)	915 (7.17)	.88
Alcohol use, No. (%)				.99
Never	7994 (31.43)	3977 (31.31)	4017 (31.55)	
Rarely to weekly	1909 (7.50)	941 (7.41)	968 (7.60)	
1-6/wk	8896 (34.97)	4511 (35.51)	4385 (34.44)	
Daily	6638 (26.10)	3274 (25.77)	3364 (26.42)	
Baseline multivitamin use	11406 (44.84)	5750 (45.23)	5656 (44.44)	.20
Baseline calcium intake ≤ 1200 mg/d, No. (%)^*b*^	5166 (19.97)	2627 (20.32)	2539 (19.62)	.16
Baseline vitamin D intake ≤ 800 IU/d, No. (%)^*b*^	11030 (42.63)	5497 (42.52)	5533 (42.75)	.72
In general would you say your health is:				.95
Excellent	6991 (29.06)	3507 (29.25)	3484 (28.87)	
Very good	10760 (44.72)	5335 (44.49)	5425 (44.95)	
Good	5386 (22.39)	2672 (22.28)	2714 (22.49)	
Fair	865 (3.60)	451 (3.76)	414 (3.43)	
Poor	58 (0.24)	26 (0.22)	32 (0.27)	
Milk (servings/d), mean (SD)	0.71 (0.91)	0.71 (0.89)	0.72 (0.92)	.23
Dark meat fish and canned tuna (servings/d), mean (SD)	0.15 (0.26)	0.15 (0.28)	0.15 (0.25)	.83
Other fish and seafood (servings/d)	0.16 (0.33)	0.16 (0.35)	0.16 (0.31)	.49
Vitamin D fortified food (servings/d), mean (SD)	0.63 (0.78)	0.63 (0.79)	0.64 (0.77)	.41
Total 25(OH)D, (SD) (n = 16 757), nmol/L	76.7 (25)	76.8 (25)	76.6(25)	.65
Free 25(OH)D, (SD) (n = 5131), pmol/L	15.04 (5.3)	15.03 (5.35)	15.05 (5.25)	.81

Abbreviations: 25(OH)D, 25-hydroxyvitamin D; SSRI, selective serotonin reuptake inhibitor.

^*a*^Race and ethnic groups self-reported by participants.

^*b*^Calcium supplement intake less than or equal to 1200 mg/d, vitamin D intake less than or equal to 800 IU/d.

Over a median of 5.3 years of follow up, there were 15 161 participants who reported a total of 51 260 falls.

Among participants answering the compliance question by questionnaire, adherence, defined as taking at least two-thirds of the trial capsules, was 85.9% in the vitamin D group and 84.7% in the placebo group at year 5. Among all participants including nonrespondents to questionnaires who were assumed to be noncompliant, adherence was 76.1% in the vitamin D group and 74.2% in the placebo group at year 5. In participants in the vitamin D group who had 25(OH)D levels repeated at 1-year follow-up, 25(OH)D levels increased by 40% from 74 nmol/L to 104 nmol/L (18).

### Fall outcome measures

**Figure 1.**
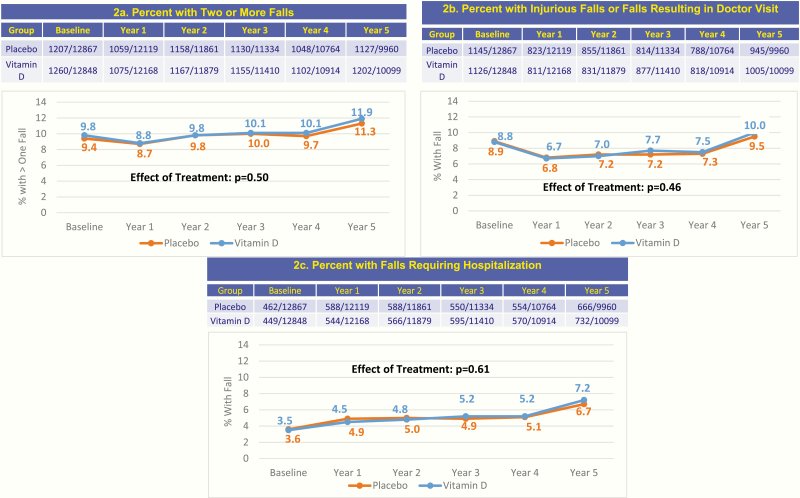
Fall outcomes over 5 years according to random assignment to vitamin D or placebo groups.

Intention-to-treat analyses demonstrated that vitamin D_3_ supplementation did not reduce falls. The number of participants with 2 or more falls, injurious falls or falls resulting in a doctor visit, and falls requiring hospitalization were similar between the vitamin D and placebo groups over the median treatment duration of 5.3 years (Fig. 1, *P* ≥ .05 for all). The odds ratio comparing the average proportions with 2 or more falls per year over 5 years of follow-up was 0.97 (95% CI = 0.90-1.05, *P* = .50), as shown in Table 2. The odds ratio for falls resulting in injury was 1.03 (95% CI = 0.94-1.13, *P* = .46), and for falls resulting in a hospital visit was 1.04 (95% CI = 0.90-1.19, *P* = .61).

**Table 2.** Percentage of participants with 2 or more falls per year by baseline characteristics

		Vitamin D		Placebo				
	No.	Baseline	Average over follow-up	Baseline	Average over follow-up	Odds ratio^*a*^ (95% CI)	*P*	*P* for interaction
Overall	25 871	11.0	11.4	10.5	11.1	0.97 (0.90-1.05)	.50	
Sex								.56
** **Men	12 687	7.9	9.3	7.5	8.8	1.00 (0.88-1.14)	.97	
** **Women	13 028	14.1	13.5	13.4	13.4	0.95 (0.86-1.05)	.36	
Age, y								.56
** **50-54	954	6.3	6.5	6.5	5.1	1.31 (0.82-2.09)	.26	
** **55-64	8791	10.0	8.6	9.5	9.0	0.91 (0.79-1.05)	.22	
** **65-74	12 664	11.4	12.3	10.3	11.5	0.97 (0.86-1.08)	.53	
** **75+	3306	13.7	17.0	14.9	17.3	1.06 (0.88-1.29)	.53	
Race/Ethnicity								.63
** **Non-Hispanic white	17 973	11.1	12.1	10.4	11.6	0.98 (0.89-1.07)	.62	
** **African American	5054	11.2	9.2	10.4	9.0	0.95 (0.79-1.14)	.59	
** **Hispanic	999	9.5	9.3	10.5	11.0	0.92 (0.62-1.39)	.70	
** **Asian/Pacific Islander	382	2.6	5.3	7.6	6.6	2.35 (0.77-7.17)	.13	
** **American Indian/Alaskan Native	226	18.8	19.2	12.7	16.9	0.77 (0.38-1.56)	.47	
Other/Unknown	517	12.8	15.3	11.8	13.9	1.02 (0.62-1.68)	.95	
BMI, kg/m^2^								.07
** **< 25	7669	10.9	10.6	9.7	10.8	0.88 (0.76-1.02)	.09	
** **25-< 30	10 032	9.1	9.6	8.5	9.0	1.00 (0.87-1.14)	.97	
** **≥ 30	7401	13.3	14.3	13.9	14.2	1.05 (0.92-1.20)	.47	
Baseline 25(OH)D								.80
** **< Median (77.5 nmol/L)	8363	11.2	11.5	10.5	11.2	0.96 (0.84-1.10)	.57	
** **≥ Median (77.5 nmol/L)	8297	11.7	12.2	10.4	11.6	0.94 (0.82-1.07)	.35	
History of hypertension								.87
** **Yes	13 269	12.3	12.8	11.9	12.7	0.98 (0.88-1.09)	.70	
** **No	12 307	9.6	9.8	9.0	9.5	0.97 (0.86-1.09)	.60	
History of diabetes								.63
** **Yes	3522	15.2	16.8	15.9	17.7	1.00 (0.84-1.19)	.99	
** **No	22 150	10.4	10.6	9.6	10.2	0.96 (0.88-1.05)	.40	
Alcohol use								.67
** **Never	7994	12.9	13.3	11.9	12.9	0.94 (0.83-1.07)	.38	
** **Rarely-< weekly	1909	10.9	11.9	12.9	11.5	1.22 (0.92-1.62)	.17	
** **1-6/wk	8896	10.2	10.5	9.8	10.2	0.99 (0.87-1.14)	.92	
** **Daily	6638	10.0	10.3	8.9	10.3	0.89 (0.76-1.05)	.18	
General health								.38
** **Excellent	6953	7.2	7.4	6.9	7.4	0.95 (0.79-1.14)	.55	
** **Very good	10 700	10.3	10.7	9.0	9.9	0.94 (0.83-1.07)	.37	
** **Good	5353	14.2	16.2	14.8	16.1	1.05 (0.90-1.22)	.54	
** **Fair or poor	918	32.0	30.6	33.4	32.7	0.98 (0.74-1.30)	.89	
Omega-3 randomization								.54
** **Active	12 860	10.6	10.9	10.0	10.8	0.95 (0.85-1.06)	.35	
** **Placebo	12 855	11.4	11.9	10.9	11.4	1.00 (0.89-1.11)	.94	

Abbreviations: 25(OH)D, 25-hydroxyvitamin D; BMI, body mass index.

^*a*^Ratio of odds ratio comparing the average percentage with falls over follow-up compared to baseline in the vitamin D vs placebo groups.

### Fall outcome measures: exploratory subgroup analyses

In exploratory subgroup analyses, the effects of vitamin D_3_ supplementation vs placebo on 2 or more falls, injurious falls or falls resulting in a doctor visit, or falls resulting in a hospital visit did not significantly differ by age, sex, race/ethnicity, or baseline BMI (data not shown).

There were also no differences in fall measures between the vitamin D and placebo groups based on stratification by these clinically relevant baseline 25(OH)D cut points <30, 31–50, 51–75, and 76–125, and >125 nmol/L (Table 3) or by baseline 25(OH)D quintiles at the following levels: 57.5, 70, 82.5, and 95 nmol/L (data not shown).

**Table 3.** Percentage with 2 or more falls per year stratified by baseline 25-hydroxyvitamin D levels over 5 years

		Vitamin D		Placebo				
Baseline 25(OH)D, nmol/L	No.	Baseline	Average follow-up	Baseline	Average follow-up	Odds ratio (95% CI)	*P*	*P* for interaction
≤ 30	545	13.9	13.7	9.4	9.0	1.03 (0.59-1.79)	.92	.34
31-50	1916	11.5	12.0	12.2	11.3	1.13 (0.87-1.48)	.36	
51-75	5846	10.9	11.1	10.0	11.3	0.91 (0.77-1.07)	.25	
76-125	7900	11.7	12.2	10.7	11.7	0.96 (0.83-1.09)	.51	
> 125	453	11.9	13.5	5.5	9.8	0.63 (0.31-1.28)	.20	

Abbreviation: 25(OH)D, 25-hydroxyvitamin D.

**Figure 2.**
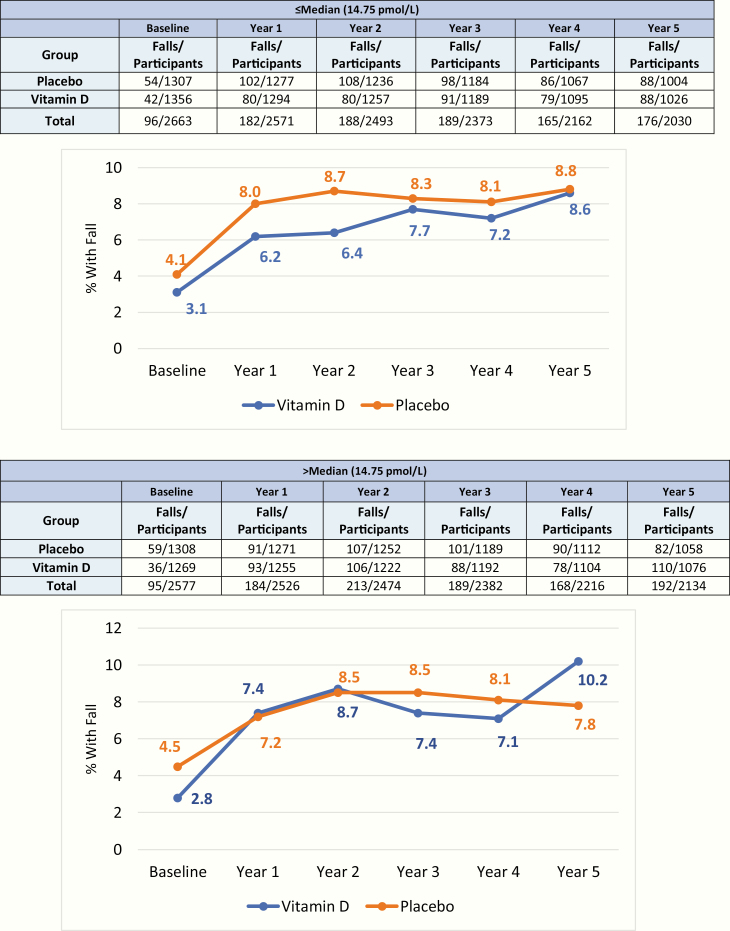
Percentage of falls resulting in a hospital visit, in those with baseline free 25-hydroxyvitamin D above and below the median (14.75 pmol/L).

Stratification by baseline free 25(OH)D levels above the median (14.75 pmol/L) demonstrated an increase in hospitalizations due to a fall in the vitamin D group compared to the placebo (*P* for treatment effect = .024, *P* for treatment × time interaction = .050, Fig. 2). This was not seen in participants with 2 or more falls or falls resulting in a doctor visit (data not shown). There were no differences in fall outcomes in those with baseline free 25(OH)D levels below the median.

Among participants with no falls at baseline, 1 fall at baseline, or 2 or more falls at baseline, vitamin D_3_ supplementation vs placebo did not affect subsequent falls, injurious falls or those resulting in doctor visits, or falls resulting in a hospital visit over 5 years (*P *> .05 for all categories; data not shown). There was also no effect of supplemental vitamin D_3_ vs placebo on fall outcomes based on those who were compliant.

### Physical activity outcome measures

In exploratory analyses, there were no differences in MET hours between the vitamin D (23.87 h) and placebo (23.95 h) groups at 3 years (*P* = .82, data not shown). There were also no differences in ADL, such as feeding or dressing oneself, and IADL, such as carrying groceries or walking more than 1 mile, between the 2 groups at baseline and year 5 (*P *> .05 for all categories, data not shown). There was no overall or average effect over time of these ADL on any of the fall outcomes (data not shown).

## Discussion

Daily vitamin D_3_ supplementation did not reduce the risk of 2 or more falls, injurious falls, or falls resulting in a doctor or hospital visit compared with placebo in generally healthy, community-dwelling older adults in the United States not selected for vitamin D insufficiency. In exploratory analyses, stratification by baseline 25(OH)D levels did not affect fall risk, including at very low levels (< 30 nmol/L) or at high levels (> 125 nmol/L).

VITAL is the largest placebo-controlled RCT of supplemental vitamin D in the United States, with a long study duration and an oversampling of African Americans. Additionally, this is one of the first RCTs to explore whether effects of supplemental vitamin D on falls are modified by baseline total and free 25(OH)D levels. Whether supplemental vitamin D in those with baseline free 25(OH)D levels above the median may be harmful in terms of falls resulting in hospitalizations warrants further study.

The findings of VITAL confirmed other RCTs that vitamin D_3_ supplementation does not reduce falls. In the recently completed Vitamin D Assessment Study in New Zealand, a placebo-controlled RCT, bolus dosing of 100 000 IU monthly over 4 years did not prevent falls in healthy adults with a mean age of 65.9 years and baseline total 25(OH)D level of 63 nmol/L (27). Another placebo-controlled RCT of community-dwelling women age 70 to 80 years and with a baseline total 25(OH)D level of 32 nmol/L in Finland investigating effects of supplemental vitamin D (800 IU/d) with or without exercise found no effect of vitamin D on the rate of falls (28). Other RCTs with positive and negative findings were likely due to differences in baseline 25(OH)D levels, patient population, and dosing. A 1-year dose-ranging study in Nebraska in postmenopausal women selected for 25(OH)D levels less than 50 nmol/L indicated in a secondary analysis a U-shaped curve of effects of combined vitamin D doses on fall risk, compared with a placebo group. However, this study was not powered to test effects of each of the 7 doses of vitamin D on falls (29). Annual bolus doses of 500 000 IU and monthly bolus doses of 60 000 IU have been shown in prior studies to increase falls in community-dwelling older adults age 70 years or older (15, 16). In our large VITAL study, the baseline mean serum total 25(OH)D level was likely sufficient for musculoskeletal health at 77 nmol/L. We found no evidence that the effect of vitamin D supplementation on fall risk varied across baseline total 25(OH)D levels ranging from less than 30 nmol/L to greater than 125 nmol/L in the general United States population, but there was a potential signal for increased falls resulting in hospitalization in those with high free 25(OH)D levels above the median.

Mechanistically, observational studies suggest associations between vitamin D deficiency and loss of type II muscle fibers, atrophy of proximal muscles, impaired muscle function, limited physical activity, and an increased risk of falling, and an in vivo study has found age-related decrease in 1,25-dihydroxyvitamin D muscle tissue receptors (30, 31). Vitamin D_3_ supplementation has also been found to increase muscle fiber number and size in some small studies (32). The mechanisms underlying the detrimental effects of high doses of supplemental vitamin D on increased fall risk are not known; higher levels of physical activity or muscle-related changes have been explored, but did not explain these findings (15, 16).

Many lifestyle variables (decreased physical function and performance), medications (antidepressants, antihypertensive medications among others), and medical conditions (eg, poor vision, arthritis, and neurodegenerative) are associated with increased risk of falls (3, 33, 34). In this VITAL study, among the fallers, physical activity, ADL, and neurological and visual disorders and history of fractures were balanced in the placebo and treatment groups. Thus, the data from this large, rigorous randomized study suggest that previously published observational reports about the associations of vitamin D with fall-related outcomes may have been due to reverse causation and confounding influence of factors not considered in those analyses.

This trial has many strengths, including being the largest study of supplemental vitamin D compared with placebo, extensive geographic diversity with participants from 50 states, oversampling of African Americans (20%), high adherence (85.9% vitamin D group, 84.8% placebo group), and a long median study duration of 5.3 years. There were baseline and follow-up blood samples for measurements of total and free 25(OH)D levels. Vitamin D measurements were calibrated to Centers for Disease Control standards. There were no significant differences in the risks of adverse events, including hypercalcemia or kidney stones, in the vitamin D_3_ group compared to the placebo group (18). This study also had limitations. Incident falls may have been underestimated because falls were assessed annually by questionnaires only and were not adjudicated (35). There is currently no standard program for measuring free vitamin D levels, and there could be potential bias from assay variation. Further, vitamin D was administered at only one dose. Owing the community-dwelling population in this trial, the results may not be generalizable to women and men in nursing homes or residential communities.

In conclusion, in this large VITAL RCT, supplementation with 2000 IU/day of vitamin D_3_ over 5.3 years did not reduce risk of falls in generally healthy older men and women not selected for vitamin D insufficiency.

## Abbreviations

25(OH)D: 25-hydroxyvitamin D
ADL: activities of daily living
BMI: body mass index
IADL: instrumental activities of daily living
MET: metabolic equivalent of task
RCT: randomized controlled trial
VITAL: VITamin D and OmegA-3 TriaL

## Additional Information

***Disclosure Summary***: M.S.L. reports grants from the NIH during the conduct of the study. P.C. reports grants from the NIH, Abbott Nutrition, and Nestle to her institution during the conduct of the study, unrelated to the present work. N.R.C. reports grant support from the NIH during the conduct of the study. J.E.B. reports grants from the NIH; nonfinancial support from Pharmavite LLC of Northridge, California; nonfinancial support from Pronova BioPharma of Norway and BASF; nonfinancial support from Quest Diagnostics (San Juan Capistrano, California) during the conduct of the study; and other from Pharmavite (spouse on scientific advisory board), outside the submitted work. V.B. reports grants from the NIH during the conduct of the study. J.E.M. reports grants from the NIH; nonfinancial support from Pharmavite LLC of Northridge, California; nonfinancial support from Pronova BioPharma of Norway and BASF; and nonfinancial support from Quest Diagnostics (San Juan Capistrano, California) during the conduct of the study. E.M.M., S.H.C., and G.K. have nothing to disclose.

***Data Availability:*** All data generated or analyzed during this study are included in this published article or in the data repositories listed in References.
